# Effects of Thioglycolic Acid on Parthenogenetic Activation of *Xenopus* Oocytes

**DOI:** 10.1371/journal.pone.0016220

**Published:** 2011-01-31

**Authors:** Zhuoran Wang, Xiaomei Ren, Dong Wang, Yongmei Guan, Lei Xia

**Affiliations:** 1 Department of Reproductive Medicine, The First Affiliated Hospital of Harbin Medical University, Harbin, China; 2 Department of Nutrition and Food Hygiene, School of Public Health, Harbin Medical University, Harbin, China; 3 School of Public Health, Mudanjiang Medical College, Mudanjiang, China; University of Colorado at Boulder, United States of America

## Abstract

**Background:**

Existing in Permanent-wave solutions (PWS), thioglycolic acid (TGA) is widely used in hairdressing industry for its contribution to hair styling. However, the toxicity of TGA, especially its reproductive toxicity, gradually calls the attention of more and more researchers.

**Method:**

In this work, *xenopus* oocytes were pretreated with different concentration of TGA, and then activated by calcium ionophore A23187. During culture, the oocytes activation rates were taken note at different time after adding calcium ionophore A23187. At the end of the culture period, the nuclear status was detected under confocal microscope. In addition, some other samples were collected for Western-Blotting analysis.

**Result:**

TGA significantly inhibited the oocytes activation rate and pronuclear formation. It may be resulted from the inhibition of the degradation of p-ERK1, Mos and CyclinB2.

**Conclusion:**

TGA inhibits in vitro parthenogenetic activation of *xenopus* oocytes with inhibited the degradation of proteins involved in mitogenic-activated protein kinase (MAPK) and maturation-promoting factor (MPF) pathways.

## Introduction

Stimulation of progesterone on *xenopus* oocytes which arrest in germinal vesicle (GV) stage induces oocytes germinal vesicle breakdown (GVBD) and the emitting of first polar body (PB1). After GVBD, oocytes enter and then arrest in the second meiosis metaphase (MII) until fertilization or parthenogenetic activation, which means MII-arrested eggs activated by physical or chemical factors, such as electricity, ethanol, 6-Dimethylaminopurine (6-DMAP), calcium ionophore A23187, Strontium Chloride (SrCl_2_), and so on. After fertilization or parthenogenetic activation, MII-arrest eggs release from this block and continue the development, release the second polar body (PB2). Parthenogenetic activation is an extremely similar process as fertilization, thus, the study on parthenogenetic activation is an excellent model for fertilization, and this can do a favor to the analysis of the failure of human oocytes fertilization and make the etiological diagnosis for person with infertilitas feminis.

The process of oocytes activation is complicated and precisely regulated by various signal pathways. Among the complicated network, maturation-promoting factor (MPF) and mitogenic-activated protein kinase (MAPK) play very significant roles during this procedure. The breakthrough from the metaphase arrest of oocytes depends on the breakdown of MPF activity, which is correlated with cytostatic factor (CSF). MAPK could mediate the CSF activity of p39 mos by preventing the cyclin degradation pathway from being turned on[1]. It has also been reported that p90 rsk was an essential mediator of CSF activity in *xenopus* eggs[2]; however, converse conclusion was got in mouse oocytes[3], this may result from the species differences.

Just due to the complexity and demanded precision during the developmental process of cccytes, exogenous chemicals always directly or indirectly impact on this procedure. Thioglycolic acid (TGA) is a major active ingredient of permanent waving solution (PWS). It has been reported that mice treated with TGA either through skin intact or inhalation might have some inhibit effect on the humoral immunity and nonspecific immunity[4], [5]. Moreover, persistent exposure to PWS was considered to lead to higher risk of menstrual disorder, spontaneous abortion and infertility in women[6]. Our teammates have confirmed that TGA can delay the GVBD of *xenopus* oocytes and inhibit its maturation induced by progesterone[7]. Based on these results, the purpose of present study is to investigate the effect of TGA on the parthenogenetic activation of *xenopus* oocytes induced by calcium ionophore A23187 that will give a further understanding for its reproductive toxicities.

## Materials and Methods

### Preparation and handling of oocytes

Adult *xenopus* laevis females were purchased from Maoshen Biotech (Shanghai, China) and maintained under laboratory condition. One week ahead of obtaining ovaries, the females were given an injection of 50IU pregnant mare serum gonadotropin (PMSG). Ovarian clumps were surgically removed from the *xenopus* laevis. The eggs we got from the ovaries were enclosed in follicle envelops. To get free eggs, we treated the ovarian clumps in Ca^2+^-free ND96 [8](96 mM NaCl, 2 mM KCl, 1 mM MgCl_2_, 5 mM HEPES, pH 7.4) containing 2 g/L Collagenase type I (Sigma) for 1–2 h, at the room temperature, and then transferred into and rinsed in MMR[9](100 mM NaCl, 2 mM KCl, 1 mM MgCl_2_, 2 mM CaCl_2_, 0.1 mM EGTA, 5 mM HEPES, pH 7.7), in which all experiments were performed. Fully-grown oocytes (stage VI)[10], which looks even dark in the animal pole, were manually separated with watch-maker forceps under stereoscope and rinsed in MMR for many times. Progesterone (Sigma) was made as a 5 mg/ml stock solution in dimethyl sulfoxide (DMSO). The maturation is induced by progesterone at a final concentration of 0.2 mg/ml, room temperature, and overnight. Mature oocytes were picked out and treated with different does of TGA (0, 5, 25, 125 µg/ml) for 2 h. After rinsing off the TGA with MMR, the oocytes were subsequently activated by adding calcium ionophore A23187 (Sigma) into MMR buffer at a final concentration of 2 µg/ml.

### Cytologicla observation

According to previous studies by Becker[11], immunofluorescence of *Xenopus* oocytes was performed. Oocytes were respectively collected 1 h after the adding of calcium ionophore A23187. This step must be performed speedily and the collected oocytes should be rapidly rinsed in MMR for at least 3 times to stop the continue action of A23187. (1) Fixation: oocytes were fixed in 3.7% formalin for 2–4 h at room temperature or overnight at 4°C. Subsequently, the oocytes were postfixed in 100% methanol overnight at –20°C. (2)Hemisection: rehydrated the samples in 3 consecutive 10-min rinses in 50% methanol/50% TBS (155 mM NaCl, 10 mM Tris-HCl, pH 7.4), 30% methanol/75% TBS, and 100% TBS, and hemisected the oocytes along the animal-vegetal axis. (3) Bleaching: oocytes were bleached in 10% hydrogen peroxide/67% methanol for 12–48 h at room temperature under fluorescent illumination. Then, aspirated the bleaching solution off carefully and rinsed the samples 3–4 times (1 h each) with TBSN (TBS with 0.1% Nonidet P-40). (4) Borohydride reduction: incubated the samples in TBS containing 100 m*M* NaBH4 for 4–6 h at room temperature or overnight at 4°C to reduce yolk platelet autofluorescence. After aspirating the NaBH_4_ off, wash the oocytes in several changes of TBSN for 1–3 h at room temperature. (5) Staining: incubate the samples in staining solution (10 mg/ml Hoechst 33258) overnight at room temperature (cover the samples to keep dark from this step on). (6) Clearing and mounting: after 3 rinses with TBSN, 1 h each, the oocytes were dehydrated in 3 to 4 changes of 100% methanol for 1–2 h, and then cleared with Murray's solution (benzylbenzoate:benzylalcohol = 2∶1), subsequently mounted onto depression slides. The specimens were analyzed with a Nikon TE 2000 confocal microscope.

### Antibodies, Proteins, and Western Blotting

The antibodies against *xenopus* CyclinB2 were purchased from Abcam, while the antibodies aginst Mos, phospho-ERK1 (p-ERK1) and ERK1 from Santa Cruz Biotechnology. To prepare homogenates, oocytes were crushed in 5 µl lysis buffer (50 mM Tris, 150 mM NaCl, 0.1% NP-40, 0.5% Deoxycholic acid, pH 7.4, 1 mM phenylmethyl sulfonylfluoride, 10 µg/ml protease inhibitor cocktail [Sigma]) per oocyte, and then briefly centrifuged for 15 min at 20,000 rpm. Protein homogenates from oocytes collected at indicated time after the addition of A23187 were subjected to sodium dodecyl sulfate polyacrylamide gel electrophoresis (SDS-PAGE) and Western Blotting analysis. β-actin was used as a loading control.

### Statistical Analysis

Data about activation rate was analyzed with SPSS16.0 by χ^2^ test. A *p* value less than .05 was considered significant.

## Results

### Effect of TGA on parthenogenetic activation rate of xenopus oocytes

**Figure 1:**
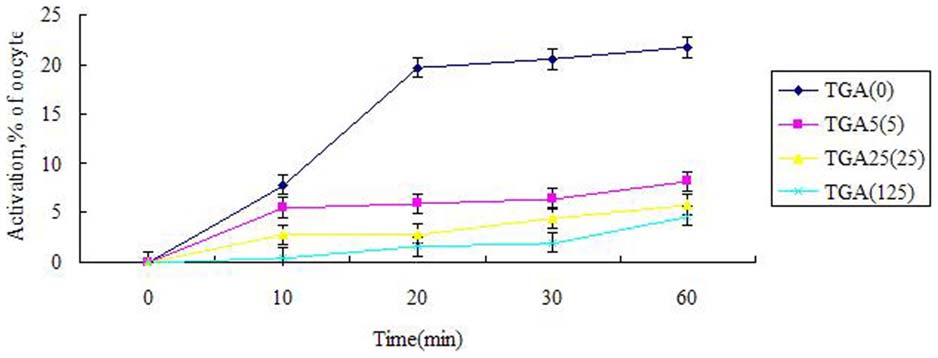
Effect of TGA on calcium ionophore A23187 induced parthenogenetic activation rate of *xenopus* oocytes in vitro. *Xenopus* MII oocytes were firstly pretreated with different dose of TGA (0, 5, 25, 125 µg/ml), after rinsed off TGA with MMR, the oocytes were then activated by 2 µg/ml calcium ionophore A23187. Oocytes activation was closely monitored after the addition of A23187. The activation rates were counted at the indicated time point (10, 20, 30, 60 min after A23187 addition).

Following TGA-pretreatment of MII oocytes for 2 h, they were activated by calcium ionophore A23187. The oocytes activation rates of indicated time were taken notes. As shown in figure 1, oocytes instantly resumed meiotic division after the addition of A23187. 10 min after the addition of A23187, the oocytes activation rate (0.51%) in 125 µg/ml TGA group was significantly lower than the control group (7.78%). Continually, compared to the control, TGA transparently inhibited the A23187 induced activation of *xenopus* oocytes in the next three check points (*P*<0.05).

### Effect of TGA on the fertilization coat formation of xenopus oocytes

**Figure 2:**
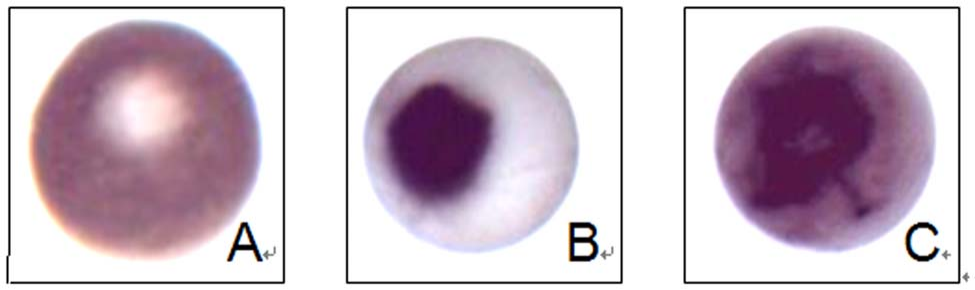
Effect of TGA on the fertilization coat formation of *xenopus* oocytes. (A) An unfertilized egg which arrested in MII, there is a white spot on the animal pole; (B) An parthenogenetic activated egg induced by 2 µg/ml calcium ionophore A23187 without TGA treatment. (C) An partheogenetic activated egg induced by 2 µg/ml calcium ionophore A23187 pretreated with 125 µg/ml TGA for 2 h.

Before activation, a white spot clearly existed on the animal pole of MII oocytes (Fig. 2A). After the addition of A23187, we found that the animal pole of oocytes gradually darkened. Finally, one fertilization coat, appeared as a black spot, can be seen on the animal pole. The spot appeared round and well-arranged in the negative control (Fig. 2B). However, the formed coat was not in good condition and appeared to be crude following TGA treatment (Fig. 2C).

#### Effect of TGA on the pronuclear formation of xenopus oocytes

**Figure 3:**
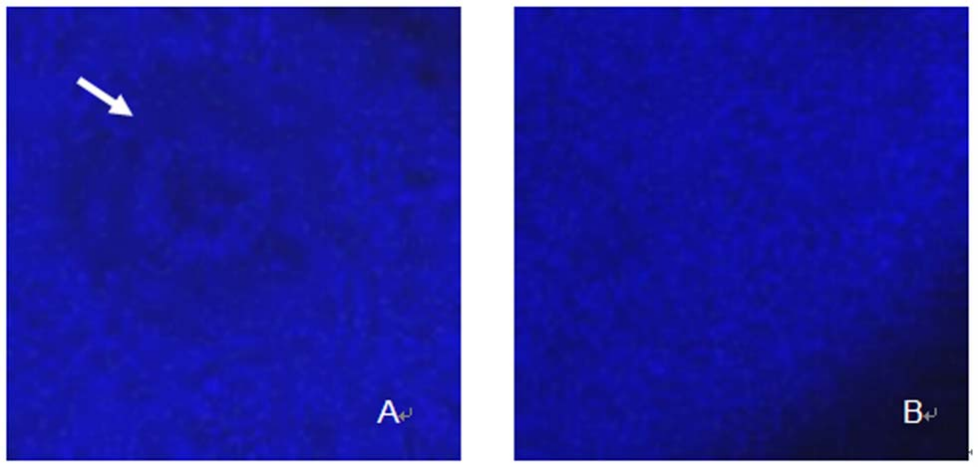
Effect of TGA on the pronuclear formation of *xenopus* oocytes. Ooytes were activated with A23187, 1 h after the addition of the stimulus, oocytes were fixed, stained with Hochest 33258 and examined under confocal microscope. (A)Pronucleus could be observed (marked by the white arrow) in the control group which was not treated with TGA. (B) Oocytes treated with 125 µg/ml TGA failed to form pronucleus.

Under confocal microscope, 1 h after the addition of A23187, a female pronucleus can be observed in the control oocytes (Fig. 3A, white arrow), which were activated by A23187 in absence of TGA treatment. In comparison with the control group, however, 125 µg/ml TGA treatment significantly leaded to the failure of the female pronuclear formation (Fig. 3B).

### Effect of TGA on the activity of MPF and MAPK

**Figure 4:**
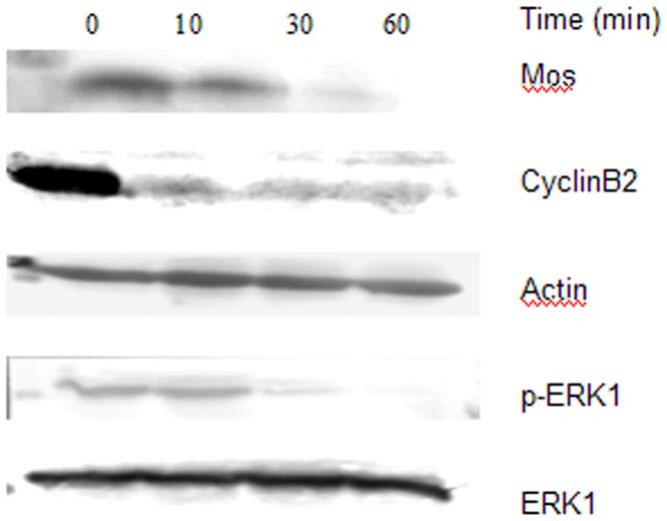
Expression of proteins involve in MPF and MAPK in *xenopus* oocytes at different time after the addition of calcium ionophore A23187 without TGA treatment.

**Figure 5:**
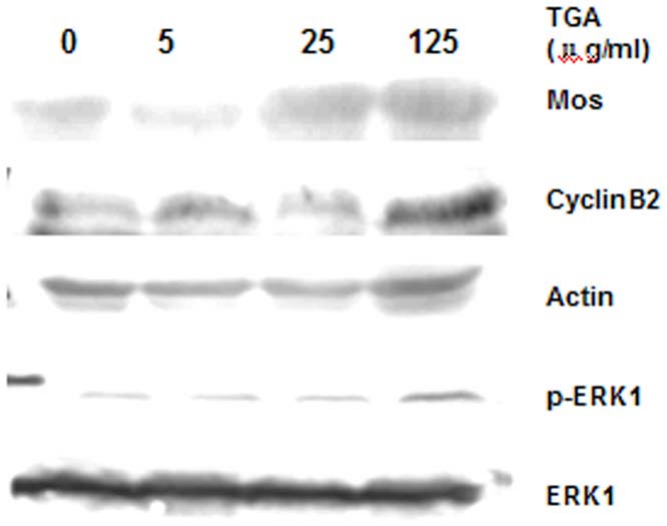
Effects of TGA on the expression of proteins involved in MPF and MAPK during the process of parthenogenetic activation. Oocytes were homogenized 1 h after calcium ionophore A23187 addition and immunoblotted with the indicated antibodies. Actin was used as a control for protein loading.

**Figure 6:**
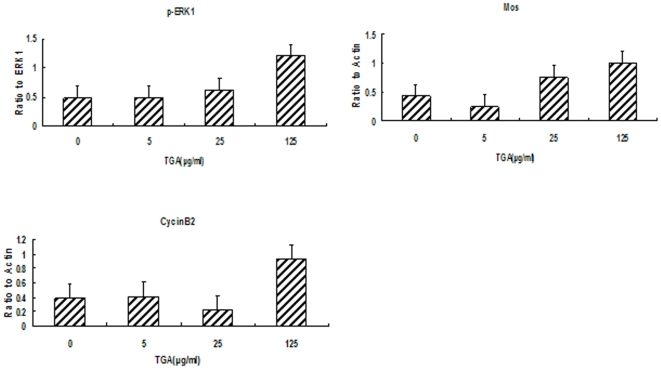
Effects of TGA on the expression of proteins involved in MPF and MAPK during the process of parthenogenetic activation. The bar graph shows the relative quantification of band intensity. Significance indicated by *p<.05, **p<.01, compared with TGA 0 µg/ml group.

To address the activation of *xenopus* oocytes, the examining on MPF and MAPK pathways is inevitable. Ahead of testing the effect of TGA on the activity of MPF and MAPK, we detected the fluctuation of MPF and MAPK at indicated time after adding the calcium ionophore A23187 (Fig. 4). CyclinB, defined as one significant element of MPF, has been detected in our test to represent the activity of MPF. As shown in figure 5 and 6, the degradation of CycinB2 was inhibited following TGA treatment. The expression of Mos and ERK1/p-ERK1 has been examined to indicate the change of MAPK activity during parthenogenetic activation following TGA treatment.

As shown in figure 4, CyclinB2, p-ERK1, and Mos degraded quickly after adding the calcium ionophore A23187, both p-ERK1 and Mos disappeared 60 min after the addition.

Based on the above results, we defined to collect oocytes 60 min after the addition of calcium ionophore A23187 to detect the effect of TGA on the activity of MPF and MAPK.

As shown in figure 5 and 6, in comparison to the control group, the expression of Mos was up-regulated in TGA 25 and 125 µg/ml, however, out of our anticipation, it was down-regulated in TGA 5 µg/ml treatment. Different from Mos, the inactivation of p-ERK1 was depressed following TGA 125 µg/ml treatment.

## Discussion

As the important ingredient of PWS, TGA is widely used in hairdressing industry. However, a surprising phenomenon called the attention of investigators that PWS long-term exposure leaded to the disturbance of female menstrual cycle and enhanced menorrhea[12]. Hairdressers always experienced a risk of fertility disorders and pregnancy complications [13]. It was also reported that hairdressers had an increased risk of prolonged time-to-pregnancy, spontaneous abortion, a low-birth weight, and more major malformations [14]. Studies on the toxicity of TGA showed that TGA can be absorbed through intact skin, inhalation, and occasionally ingestion [5], [15]. Except from hepatotoxicity and immunotoxicity, close attention has been paid to the reproductive toxicity of TGA. Studies proved that TGA was found to disturb the estrous cycle, as well as alter the normal structure of testes and ovaries of rats[15], [16]. TGA has also proved to disturb the spindle formation, chromosome alignment and delay the extrusion of PB1 during the meiotic maturation of mouse oocytes[17]. In *Xenopus* oocytes, TGA accelerated GVBD occurrence, but still delayed the extrusion of PB1, inhibited MI-MII transition, and thus finally depressed the maturation *in vitro* [18].

Germ cells are elementary for reproductive function. Well-development of oocytes ensures the further behavior of females. Fully grown *xenopus* oocytes are arrested at the first meiotic metaphase until progesterone triggers meiotic maturation. After undergoing GVBD and emitting the PB1, mature eggs enter and arrest in MII till fertilization or parthenogenetic activation.

Activation of *xenopus* oocytes can be detected in the live eggs by movement of pigments, cortical contraction and the formation of a fertilization coat[19], appearing as “caping” phenomenon. The observed spot formed after activation following TGA treatment was in a poor condition compared with the one formed on the oocytes which were activated by A23187 without TGA treatment. We supposed that is due to the disorder of pigments movement. Further work has been done to make sure the parthenogenetic activation. Under the confocal immunofluorescence microscopy, we observed that the oocytes failed to form pronucleus following TGA 125 µg/ml treatment compared with the control group. Given that the inactivation of MAPK is necessary for nuclear envelope when parthenogenetic activation was initiated by various stimuli[20]–[22], we presumed that the failure of pronuclear formation was due to the consistent existence of MAPK. Satisfactorily, 125 µg/ml TGA treatment led to the pronuclear formation failure,and simultaneously inhibited the proteolysis of MAPK. All of these results suggested that above guesses have been correct.

MAPK, also known as extracellular signal-regulated kinase (ERK), behaving as a significant regulatory system for meiosis,apart from the formation of nuclear envelope, is also involved in the activity of cytostatic factor (CSF), which is responsible for the metaphase arrest. A lot of evidence confirmed that c-mos mRNA is highly expressed in vertebrate gonadal tissue and Mos protein is synthesized at high level during germ cells maturation[23]. However, Mos protein disappeared after fertilization, although c-mos mRNA was still detectable[24]. It has also been proved that oocytes from c-mos deficient mice can be spontaneously parthenogenetic activated without the experience of MII arrest[25]. Besides, Hideki [26]found that MAPK inhibitor U0126 could drive porcine oocytes out of metaphase arrest. Thus, we examined the expression of Mos and p-ERK1/ERK1 to address whether the inhibition of oocytes activation following TGA treatment was mediated by MAPK pathway. It has been presented to us that TGA inhibited the degradation of Mos and p-ERK1. Therefore, we supposed that it's the TGA treatment affected the Mos expression, and further affected the expression of p-ERK of MAPK cascade. On the other hand, the reduced degradation of Mos inhibited the inactivation of CSF, which induced the inhibited-degradation of Cyclin B.

CyclinB is an egulatory subunit of MPF, which also has a catalytic subunit named Cdc2 (also known as p34^cdc2^). MPF is another factor besides CSF responsible for MII arrest. Inactivation of MPF requires ubiquitin-dependent proteolysis of Cyclins [27], which is responsible for exiting from MII arrest. In *xenopus*, MPF contains both CycinB1 and CycinB2 in similar amount [28]. Since that the appearance and disappearance of MPF depend on the synthesis and degradation of cyclins, while the concentration of Cdc2 remains constant [28], we checked the expression of CyclinB2 to explain the activity of MPF. Just as our expectation, TGA treatment inhibited the proteolysis of CyclinB2, and thus inhibited the inactivation of MPF during parthenogenetic activation.

In conclusion, TGA inhibited *xenopus* oocytes parthenogenetic activation stimulated by calcium ionophore A23187. It may be due to the disorder of MAPK and MPF inactivation. Our results in this study further suggested that TGA really has the reproductive toxicity. Protection measures should be strengthened for people who contact TGA, especially the ones of childbearing age. In order to get a further recognition of the effects of TGA on parehenogenetic activation, we are going to focus on the intracellular Ca^2+^ and calmodulin, as well as spindle migration during this process.
